# Epidemiology of cow’s milk allergy: has it changed?

**DOI:** 10.1186/2045-7022-1-S1-S50

**Published:** 2011-08-12

**Authors:** Cansin Sackesen

**Affiliations:** 1 Hacettepe University School of Medicine, Pediatric Allergy and Asthma Unit, Ankara, Turkey

## 

Our knowledge about the time trend of cow’s milk allergy prevalence is very limited, however there some indirect data favoring the increase of the cow’s milk allergy prevalence. There are several studies reporting that food allergy is increased among children of all ages, both gender and different races/ethnicities. Several national health surveys in USA recently indicated that food allergy prevalence and/or awareness has increased 18% (z=3.4; p<0.01) from 1997 through 2007. Another study from UK determined that hospital admissions for food allergy rosed from 5 to 26 per million in general population, this was particularly apparent in children where rates rose sevenfold from 16 to 107 per million. There are limited studies focusing on a specific food allergy prevalence. Several studies from UK and USA focusing on peanut showed that the prevalence of peanut allergy doubled in children. However another study searching the time trends of the food-induced anaphylaxis in the pediatric emergency department indicated that the number of visits was increased not only because of peanut–induced anaphylaxis but for multiple food allergens including cow’s milk. Another study from Australia determined four-fold increase of food allergy in 0-5 year-old children from 1995 to 2006. The common triggers of food allergy in Australian children were peanut, hen’s egg, cow’s milk and treenuts and the prevalence of food allergy was increased with time for each food including cow’s milk allergy. A final study from China investigating time trends in the prevalence of food allergy in 0-2 year-old infants from 1999 to 2009 determined that the prevalence of food allergy increased more than 2.2-fold between 1999 and 2009 and the prevalence of cow’s milk allergy increased from 1.6% to 3.5% over a decade. All the studies from different parts over the world support the opinion that the prevalence of food allergy roses and this rise covers the prevalence of the cow’s milk allergy especially in children.

